# Act II of the Sunshine Act

**DOI:** 10.1371/journal.pmed.1001754

**Published:** 2014-11-04

**Authors:** Genevieve Pham-Kanter

**Affiliations:** Drexel University School of Public Health, Philadelphia, Pennsylvania, United States of America

## Abstract

To coincide with the introduction in the United States of the Sunshine Act, Genevieve Pham-Kanter discusses what we need to look for to fight hidden bias and deliberate or unconscious corruption.

*Please see later in the article for the Editors' Summary*

**Summary Points**

- The Sunshine Act will lead firms to be more forthcoming about physician payments but will also create incentives for firms to underreport and target nonphysician prescribers.
- Whether transparency leads to diminished firm influence on doctors depends crucially on whether physicians who accept payments will be penalized by the public or other parties for accepting.
- Many preconditions must be met before patients can effectively sanction doctors for receiving payments.

With the rollout of the Sunshine Act's [1] reporting system, monetary payments that pharmaceutical and medical device firms make to physicians are now available for public viewing [2]. Enacted in 2013, the Sunshine Act's Final Rule [3] requires that drug and device firms operating in the United States report, to the US federal government and for public scrutiny, almost all payments and gifts that they make to physicians that are valued above US$10. Because the Act requires firms to report a wide variety of payments—for example, payments for meals, travel, and entertainment; compensation for consulting services; compensation for serving as a speaker for a continuing medical education program; research grants—the Sunshine Act and its Open Payments reporting system reveal a range of financial transactions that had previously been dimly and incompletely known to outsiders.

This transparency in physician payments has been widely praised. Prominent critics of doctors' ties to drug companies such as Drs. Marcia Angell, Jerry Avorn, Jerome Kassirer, Steven Nissen, and the president of the American Association for Medical Ethics, Charles Rosen [4], as well as the consumer protection advocacy group Public Citizen [5], have publicly supported the Sunshine Act. So has the industry trade group Pharmaceutical Research and Manufacturers of America [6].

One might think that transparency would be difficult to oppose. Being pro-obfuscation hardly seems like a popular position. Yet, the passage of the Sunshine Act was no small legislative achievement. The Act languished for years after its initial introduction in 2007 [7],[8] until the momentum of the Affordable Care Act (ACA) created an opportune moment to fold the Sunshine Act into the ACA's set of wide-ranging health care reforms in the US [1].

But as Open Payments reporting moves forward, we should not simply put our feet up and fire up our data servers. Rather, we should look ahead and prepare for the downstream effects of the Sunshine Act and its new rules. In particular, we should think through how Open Payments is likely to affect various stakeholders—doctors, firms, and patients—in view of the central problem that the Sunshine Act was intended to solve and make preparations to monitor potential problem areas.

Good policy making is not “one and done,” that is, putting a rule in place and living happily ever after. People (and firms) adapt to regulations, and not always in good ways, and policy makers and researchers should ideally be forward-looking, putting systems in place that allow us to evaluate and refine Open Payments rules.

The Sunshine Act was set in motion by a desire to first, make public the financial inducements used by firms to persuade doctors to favor their commercial products; and second, in making these transactions public, reduce firms' influence on doctors [3],[9]. The underlying premise of the Act is that patients would respond negatively to knowing their doctor had received payments. If patients knew about and disapproved of these payments, doctors would be less willing to accept payments, and firm influence would be diminished. This was the ideal scenario envisioned by the Act.

In real life, however, other things may happen. Because patients view some types of payments more negatively than others—doctors who accept payments for travel, meals, or entertainment, for example, are rated as having lower “moral character” than those who accept money for consulting [10]—firms may respond to the Act by simply shifting around classifications but not changing the substance of their payments. Consulting in particular seems to be a catchall that does not alarm patients but could cover a wide range of financial transfers, some of which patients might view as deeply suspect if more details were revealed about these transactions.

In addition, the Sunshine Act focuses on payments made to physicians, mostly licensed medical doctors and doctors of osteopathy. Firms need not report payments made to nonphysician prescribers such as physician assistants or nurse practitioners. With ACA coverage expansions and the aging US population, the number of physician assistants and nurse practitioners and the prescribing authority of these providers are likely to increase because the supply of primary care physicians cannot, in the short run, fully meet the rising demand in many service areas [11]–[14]. Given the rapidly expanding pool of noncovered (nonreportable) prescribers, firms are likely, and rationally so, to direct more of their dollars and attention to these nonphysician health care workers.

A third important implication of the Sunshine Act is that if patients do respond negatively to physician payments, there is a strong incentive for both firms and physicians to underreport payments. Put bluntly, there is likely to be collusion to underreport transactions. Note that collusion need not be explicit; no midnight phone calls or notes in flower pots would be involved. Rather, the collusion would be tacit and not necessarily deliberate as both parties go through the reporting process. Open Payments requires that firms first submit reports of payments to the Centers for Medicare and Medicaid Services (CMS), identifying physician recipients. Physicians then review their individual reports and have a minimum of 15 days to dispute any items. If a dispute arises, firms and physicians are required to resolve disputes between themselves, with CMS playing no role in dispute resolution [3].

Tacit collusion could occur if firms submit initial payment reports that omit items or that underapproximate the original payment. Physicians may not notice any gaps or, even if they do, are unlikely to dispute the report and volunteer additional payment information. Both parties would be worse off in a world with accurate but high levels of reported payments compared to a world with inaccurate but low reported payments. Although the Final Rule specifies penalties for inaccurate reports and makes reference to audits, the likelihood of underreporting will be high unless CMS's (unfunded) auditing system is viewed as a credible threat. (Helpful hint: economic theory predicts that even infrequent audits can ensure compliance if they are random and tied to extremely large penalties [15].)

These are all possible, perhaps even likely, implications of the Sunshine Act if patients respond negatively to learning that their doctor accepts payments. Physicians may refuse payments, but in addition, firms may systematically (mis)classify ambiguous payments in their favor, firms may increasingly target nonphysician prescribers, and both firms and physicians may have a mutual interest in underreporting payments.

And what about the possibility that patients do not respond negatively to knowing about physician payments? It is important to think through the exact mechanism through which transparency can curb firms' payments to doctors. Sunshine Act transparency will reduce payments only if physicians who are publicly revealed to have accepted payments are sanctioned somehow for having taken money. Put differently, if the public does nothing with this information—in particular, doesn't make a doctor who accepts payments worse off than if he were to reject payments—transparency will have accomplished nothing, if the goal of the Sunshine Act is to reduce payments. In sum, doctors would need to be punished, broadly speaking, for having been revealed as accepting certain payments in order for transparency in payments to work.

Two key questions are, who is going to be doing this (broadly defined) sanctioning of doctors who receive payments and how will they do it? When we talk about “the public” knowing about payments, we typically mean patients, but the ability of patients to sanction or punish doctors may be rather limited. Think about what this mechanism would involve: a patient who is informed that his or her doctor has accepted drug company money would be repelled by that doctor accepting payments, lose faith in that doctor's judgment, and then go to a different doctor who does not accept payments. Doctors, given the possibility of losing credibility as well as patients if they accept payments, would then opt to reject payments.

Revisiting these steps more carefully, we see that, first, the patient must be sufficiently repelled at the thought of a doctor receiving payment. Although patients respond negatively to some forms of payment [10], it is unclear whether all or even most patients would be put off by this knowledge. Indeed, some patients may view their doctors having been asked to serve on an advisory board or having been paid to give a talk as a sign of the expertise of their doctor. Receiving money for recruiting patients for a trial, even a trial that has minimal research validity and in many cases whose validity the patient would not be in a position to evaluate, may be viewed as a signal of being a very good doctor and not a very bad or unethical one.

Next, the patient must lose faith in the doctor's judgment. Especially for a patient who has had a long-standing relationship with a doctor and who has had years of previous aches and pains and ills apparently treated by this doctor, is it reasonable to think that this additional line item of knowledge will undo years of prior experience with and trust in the clinician? The patient must weigh this iota of new knowledge about the doctor against all of his or her previous patient experience. (Of course, this new knowledge could lead a subset of patients to completely reassess the motives of their doctors and unduly mistrust them and mistrust medical advice in general. The degree to which patient trust is actually undermined by disclosure will be important to monitor.)

Finally, the patient must be able to change doctors relatively easily. There are, of course, well-known network limits within US health care plans; patients may thus be restricted in the set of providers to which they have access. But in addition, there are search costs of finding a new doctor whom patients like and trust. Finding the right doctor takes time and effort and involves risk in trying out poorly matched doctors. The overall time and effort costs involved in switching may be too large even if patients are disappointed to find out that their doctor has received drug company payments. In short, patients can't always leave even if they don't like the fact that a doctor accepts payments from a drug or device firm.

In terms of sanctioning, then, the Sunshine Act may be asking for patients to do too much. There are other parties in the health care system who can act to penalize doctors for accepting payments. Insurers, for example, could decide to reimburse only doctors who do not accept payments because those who do accept them may have prescribing habits that are too costly. Insurers would have sufficient bargaining power vis-à-vis doctors to require that doctors not accept payments or not accept certain kinds of payments. In addition, physicians who do not accept payments could sanction physicians who do, although it is hard to see how or why this kind of sanctioning would emerge spontaneously.

Yet, recall why sanctioning came up in the first place: sanctioning, or associating some cost with receiving payments, is critical to the Sunshine Act achieving its original aim. If transparency in payments is to have some effect in curbing firm payments and firm influence (assuming that payments equal influence), we need to create a system in which doctors will be better off refusing payments rather than accepting them. In other words, a new improved Sunshine Act has to include a stick for accepting payments and bountiful carrots for not accepting payments. Historical experience tells us that disclosure without sanctioning mechanisms leads us to transparency without teeth (see, for example, the entire history of political campaign finance disclosures in the US).

This analysis has highlighted some areas that require closer scrutiny: reclassification of ambiguous payments, shifts toward nonphysician prescribers, and underreporting of transactions. The nature of these adaptations to the Sunshine Act—away from sunshine and towards the shadows, away from observable transactions and towards transactions less easily tracked—means that data from Open Payments alone will not be sufficient to evaluate the effects of the Act. An observed decrease in payments could happen because of a true decline in payments or because of, say, firms shifting to nonreportable prescribers. Additional data from other sources, such as random audits, surveys of nonphysician and physician prescribers, conflict of interest disclosures filed by employees of academic medical centers and authors of journal publications, and data from public financial filings of firms, can be used to externally validate the scope and scale of official reported transactions.

Transparency and the Sunshine Act are good achievements. Open Payments will provide much more information on physician–industry financial relationships than we had before. We will all adapt to and learn from the Sunshine Act, and it is precisely for this reason that it is not too early to begin thinking about Act II.
